# Role of PI3K/AKT pathway in squamous cell carcinoma with an especial focus on head and neck cancers

**DOI:** 10.1186/s12935-022-02676-x

**Published:** 2022-08-13

**Authors:** Soudeh Ghafouri-Fard, Ali Noie Alamdari, Yashar Noee Alamdari, Atefe Abak, Bashdar Mahmud Hussen, Mohammad Taheri, Elena Jamali

**Affiliations:** 1 Department of Medical Genetics, School of Medicine, Shahid Beheshti University of Medical Sciences, Tehran, Iran; 2 Faculty of Dentistry, Tabriz University of Medical Sciences, Tabriz, Iran; 3 Department of Pharmacology, Tabriz University of Medical Sciences, Tabriz, Iran; 4 Men’s Health and Reproductive Health Research Center, Shahid Beheshti University of Medical Sciences, Tehran, Iran; 5 Department of Pharmacognosy, College of Pharmacy, Hawler Medical University, Kurdistan Region, Erbil, Iraq; 6 Center of Research and Strategic Studies, Lebanese French University, Kurdistan Region, Erbil, Iraq; 7 Institute of Human Genetics, Jena University Hospital, Jena, Germany; 8 Urology and Nephrology Research Center, Shahid Beheshti University of Medical Sciences, Tehran, Iran; 9 Department of Pathology, Loghman Hakim Hospital, Shahid Beheshti University of Medical Sciences, Tehran, Iran

**Keywords:** PI3K/AKT pathway, Squamous cell carcinoma

## Abstract

PI3K/AKT pathway is an important pathway in the carcinogenesis since it has central impacts in the regulation of metabolic pathways, cell proliferation and survival, gene expression and protein synthesis. This pathway has been reported to be dysregulated in several types of cancers. In the current review, we summarize the role of this signaling pathway in squamous cell carcinomas (SCCs) originated from different parts of body cervix, oral cavity, head and neck and skin. The data presented in the current review shows the impact of dysregulation of PI3K/AKT pathway in survival of patients with SCC. Moreover, targeted therapies against this pathway have been found to be effective in reduction of tumor burden both in animal models and clinical settings. Finally, a number of molecules that regulate PI3K/AKT pathway can be used as diagnostic markers for different types of SCCs.

## Introduction

PI3K/AKT pathway has important roles in the carcinogenesis since it has central impact in the regulation of metabolic pathways, cell proliferation and survival, gene expression and protein synthesis [1]. As a multimember family of heterodimeric lipid kinases, PI3Ks are classified into three distinct classes. Class IA PI3Ks are induced by receptor tyrosine kinases such as p110 catalytic subunit as well as p85-like regulatory subunits [1]. Class IB PI3Ks are induced by G protein-coupled receptors and regulatory subunits. Class II PI3Ks includes three proteins, namely PIK3C2A, PIK3C2B and PIK3C2G. Finally PIK3C3 is regarded as the single member of class III PI3Ks. PI3Ks can be induced by several upstream cell-surface receptors. In response to these stimuli, class I proteins catalyze the conversion of PI(4,5)P2 to the second messenger PIP3. AKT and PDK-1 serine/threonine kinases are two proteins that have PIP3-binding Pleckstrin homology (PH) domain and are associated with PI3K in a variety of cells [2, 3]. AKT is an evolutionarily conserved serine protein kinase being attributed to the AGC subfamily. This protein has three structural domains, namely N-terminal PH domain, a short C-terminal tail comprising a regulatory hydrophobic motif (HM) and a linker section with a central kinase catalytic domain [6]. AKT family of proteins includes three homologous subtypes, namely AKT1-AKT3. In response to increase in PI(3,4,5)P3 levels and to a lesser extent accumulation of PI(3, 4)P2, AKT is recruited on the cell membrane through its PH domain and exerts its catalytic roles through activation of a PDK1-induced threonine phosphorylation and mTORC2-mediated serine phosphorylation. These phosphorylation events occur at specific sites of AKT1, AKT2 and AKT3 [4, 5]. The effects of AKT on regulation of important downstream effectors including FOXO, mTOR and GSK3b endows this molecule the ability to influence cell proliferation and survival, genome stability, and metabolic pathways [1]. PI3K/AKT pathway has been reported to be dysregulated in several types of cancers. In the current review, we summarize the role of this signaling pathway in squamous cell carcinomas (SCCs) originated from different parts of body cervix, oral cavity, head and neck and skin.

## Cervical cancer

Hou et al. have assessed the clinical outcomes of individuals with metastatic or recurrent cervical cancer during a phase I clinical trial. They have reported longer survival of patients with SCC of the cervix who had PIK3CA mutations compared with those without PIK3CA mutations. In fact, their results have shown that matched therapies against the activated PI3K/AKT/mTOR pathway have significant clinical benefit [6]. Another study in the context of cervical SCC has shown over-expression of the endogenous inhibitor of mTOR complexes DEPTOR in these cells and tissues. DEPTOR silencing has enhanced apoptosis of these cells via increasing expression of p38 MAPK and suppression of PI3K/AKT activity through feed-back suppression from mTORC1-S6K. Moreover, knock down of this gene has led to reduction of levels of nitric oxide synthases iNOS and eNOS, and enhancement of activity of ERK1/2 and p38 MAPKs. Moreover, DEPTOR could affect ERK1/2 expression in through modulation of AKT. Cumulatively, DEPTOR increases survival of cervical SCC cells and its knock down leads to cell apoptosis through distinctive impacts on PI3K/AKT and p38 MAPK [7]. Moreover, the over-expressed receptor for advanced glycation endproducts (RAGE) has been shown to be involved in the pathogenesis of cervical SCC through modulation of PI3K/AKT activity. This protein has been found to promote proliferation of cervical SCC, enhance expression of PCNA, inhibit cell apoptosis along, reduce Bax/Bcl-2 ratio, and induce activity of PI3K/AKT pathway. RAGE silencing has reduced tumor burden in a xenograft model of cervical SCC. Finally, the PI3K inhibitor LY294002 could efficiently inhibit activity of PI3K and AKT, and suppress RAGE-induced pro-proliferative and anti-apoptosis effects [8]. Table 1 shows the role of PI3K/AKT pathway in squamous cell carcinoma of cervix.

**Table 1:** Role of PI3K/AKT pathway in squamous cell carcinoma of cervix

Samples	Cell lines	Drug/phytotherapy	Dose range	Target	Pathway	Function	Refs.
Metastatic or recurrent cervical SCC (n = 31)	–	–	–	–	–	Targeted PI3K/AKT/mTOR therapies in patients with heavily treated metastatic or recurrent cervical SCC who harbor PIK3CA mutation and/or PTEN loss/mutation are associated with a significant response rate and survival benefits	[6]
–	SiHa, ME-180, HeLa, C33A	DEPTOR siRNA	90 nM	Bcl-2, Bcl-xL	PI3K/AKT, p38 MAPK, ERK1/2	DEPTOR silencing via down-regulating PI3K/AKT and by up-regulating p38 MAPK could induce apoptosis	[7]
–	SiHa, CaSki, C33A, MS751	FPS-ZM1	1 μM	RAGE, Bax, Bcl-2, PCNA	PI3K/AKT	Downregulation of RAGE via modulation of PI3K/AKT can activate apoptosis and inhibit cell proliferation in cervical SCC	[8]
Primary cervical cancer (n = 70), normal cervical tissues (n = 30)	HeLa, SiHa, ME-180, CaSki, C-33A, C-4I, SW756, MS751	–	–	p27Kip1, AKT1	PI3K/AKT	Downregulation of p27(Kip1) could be regulated via the PI3K/AKT-mediated proteasomal degradation in CC cells	[9]
Primary CC (n = 35), normal cervical tissues (n = 35)	HeLa, CaSki, SiHa, ME-180, H8	–	–	ANRIL	PI3K/AKT	LncRNA ANRIL could promote carcinogenesis via PI3K/Akt pathway and can be considered as an indicator of poor prognosis	[10]
-	HeLa	Nicotine	0.1–10 μM	NF-κB	PI3K/AKT	Nicotine via induction of PI3k/AKT/NF-κB pathway promotes HeLa cell migration and invasiveness	[11]
Primary CC (n = 93)	Hela, Caski	LY294002, cisplatin	10–30 nM, 10 μM	PAK4	PI3K/AKT	PAK4 via the PI3K/AKT pathway can contribute to the cisplatin resistance in CC cells	[12]
Primary CC (n = 136)	HeLa	–	–	PGRN, TSC-2, p70S6K	PI3K/AKT, mTOR, ERK	Growth factor progranulin (PGRN) via the PI3K/AKT/mTOR pathway can promote tumorigenesis of CC	[13]
Primary CC (n = 219)	HeLa, ME-180, SiHa, C33A, CaSKi, MS751	–	–	FOXC1	PI3K/AKT	FOXC1 via the PI3K-AKT signal pathway can promote proliferation and EMT in CC	[14]
Primary cervical cancer (n = 174), healthy volunteers (n = 30)	–	–	–	–	PI3K/AKT, mTOR	Exosome-mediated PI3k/Akt/mTOR pathway could be considered as a diagnostic biomarker in CC	[15]
–	SiHa, C33A, CaSki, HK-2, WI-38, HeLa	Licochalcone A (LicA)	0–100 μM	LC3-II, Beclin-1, Atg-5/7/12, Bcl-2, Caspase-3/9, JNK1/2	PI3K/AKT, mTOR	LicA via inactivating the PI3K/AKT/mTOR pathway could induce autophagy in CC cells	[16]
–	HeLa, SiHa, CaSki	–	–	S100A6, GSK-3β, E-cadherin, N-cadherin, Vimentin, Snail, Twist	PI3K/AKT, mTOR	S100A6 via the PI3K/AKT pathway promotes proliferation and migration of CC cells	[17]
Primary CC (n = 72) healthy volunteers (n = 12)	CaSki	–	–	miR-433, FAK	PI3K/AKT	miR-433 via PI3K/AKT signaling by influencing expression of FAK could induce apoptosis in CC	[18]
Primary CC (n = 30) healthy volunteers (n = 12)	Hela, C33A, SiHa, ME-180	–	–	miR-338, ATF2, LC3I/II, Bax, Cyclin-D1, p27/35, Bcl-2, Caspase-3/9	PI3K/AKT, mTOR	miR-338 via the PI3K/AKT/mTOR pathway could modulate proliferation and autophagy in CC	[19]

## Head and neck squamous cell carcinoma

### Laryngeal squamous cell carcinoma (LSCC)

Mukonal, the isolated alkaloid from the plant *Murraya koenigii* has been shown to reduce the viability of laryngeal SCC cells, induce their apoptosis and arrest them at G2/M phase possibly through suppression of activity of PI3K/AKT and MEK/ERK pathways [20]. Moreover, dehydrocostus lactone isolated from *Saussurea costus Lipech* has been found to exert cytotoxic effects in this type of cancer. This substance could inhibit viability, migration and proliferation of laryngeal SCC cells without affecting viability of normal larynx epithelial cells. Notably, dehydrocostus lactone could promote function of p53 and P21 and induce cells apoptosis through suppression of PI3K/Akt/Bad pathway and stimulation of endoplasmic reticulum stress-mediated apoptotic pathways. In vivo assays have also verified these effects [21].

Another study has shown up-regulation of FGFR1, FGFR3 and PI3K/AKT kinase expression levels in the squamous cell laryngeal cancer samples compared with non-cancerous laryngeal mucosa specimens. Notably, over-expression of PI3K/AKT kinase has been associated with a high tumor front grading. Moreover, levels of the p-PI3K regulatory kinase protein have been associated with survival rate of patients. Taken together, FGFR1, FGFR3, and downstream regulatory kinases from the PI3K/AKT pathway might be regarded as putative markers indicative of invasive properties of laryngeal cancer [22]. Table 2 shows the role of PI3K/AKT pathway in laryngeal squamous cell carcinoma.

**Table 2:** Role of PI3K/AKT pathway in laryngeal squamous cell carcinoma

Samples	Cell lines	Drug/phytotherapy	Dose range	Target	Pathway	Function	Refs.
–	AMC-HN-8, HuLa-PC	Mukonal	0–100 µM	–	PI3K/AKT, MEK, ERK	Mukonal by affecting activity of the PI3K/AKT and MEK/ERK pathways and by promoting apoptosis and G2/M cell cycle arrest could inhibit the migration/invasion and proliferation of LC cells	[20]
BALB/c nu/nu	Hep-2, TU212, HBE	Dehydrocostus Lactone (DHL)	0–10 µg/mL	Bcl-2, Bax, Bad, p53, p21, PTEN Waf1/Cip1, Cyclin-D1, MMP-2/9, Caspase-12/9/3	PI3K/AKT	DHL by stimulating endoplasmic reticulum (ESR) stress and inhibiting PI3K/AKT/Bad signaling pathway could inhibit cell proliferation	[21]
LSCC (n = 137), non-cancerous laryngeal mucosa (n = 100)	–	–	–	FGFR1, FGFR3	PI3K/AKT	FGFR1 and FGFR3 via targeting the PI3K/AKT pathway could be involved in the invasiveness and prognosis of LSCC	[22]
LSCC (n = 110), laryngeal severe dysplasia (n = 30)	AMC-HN8, TU212, TU686	–	–	FADS1, S6K1	AKT/mTOR	Overexpression of FADS1 via activating the AKT/mTOR pathway could promote LSCC growth and migration/invasion	[23]
Cohort, BALB/C nude mice	Tu 177/Cis, HOK, 293 T, MRC-5, FD-LSC-1/Cis	Cisplatin	0–25 µg/mL	miR-145-5p, circPARD3, p62, LC3B-I/II, PRKCI	AKT/mTOR	CircPARD3 through the miR-145-5p/PRKCI/AKT/mTOR axis could promote proliferation, migratory potential, invasion, and chemoresistance	[24]
LSCC (n = 53), ANM (n = 53), male BALB/C nude mice	FD-LSC-1, TU-177	–	–	SKA3, PLK1, HK2, PFKFB3, PDK1, PTEN, c-Myc	AKT	SKA3 via interacting with PLK1 to activate the AKT pathway by up-regulating glycolysis level could suppress the chemoresistance and proliferation of LSCC	[25]
–	Hep-2	–	–	SHIP2, p21, p27, Caspase-3	PI3K/AKT	Knockdown of SHIP2 could inactive the PI3K/AKT pathway. Hence, it could be involved in radiosensitivity of LSCC	[26]
BALB/cA nu/nu	AMC-HN-8			miR-145	PI3K/AKT	miR-145 via the PI3K/AKT axis can inhibit the proliferation and growth of LSCC	[27]
16 pairs of LSCC and adjacent normal tissues	AMC‐HN‐8, TU212	–	–	MMP-2/3, NF-kB, E‐cadherin, Vimentin, Occluding, N‐cadherin,	PI3K/AKT	Knockdown of MMP2/3 via the PI3K/AKT/NF-kB axis can affect proliferation and migration of LSCC cells	[28]
46 pairs of LSCC and adjacent normal tissues	Hep-2, TU212, AMC-NH-8, TU686	–	–	MEOX2, c-Myc, Caspase-3, XIAP	PI3K/AKT	MEOX2 through inhibiting the PI3K/AKT pathway can suppress cancer cell viability and apoptosis	[29]
20 pairs of LSCC and adjacent normal tissue	Hep-2	–	–	Tra2β, Bax, Bcl-2, Caspase-3	PI3K/AKT	Silencing of Tra2β via inhibiting the PI3K/AKT pathway leads to suppression of proliferation, invasiveness, and migration of malignant cells	[30]
32 pairs of LSCC and adjacent normal tissues	TU-177, TU686, TU212, AMC-HN-8, NHOKs	Curcumin	0–40 µM	miR-145	PI3K/AKT, mTOR	Curcumin via up-regulation of miR-145 and inhibiting PI3K/AKT, mTOR pathway could suppress LSCC progression	[31]
65 pairs of LSCC and adjacent normal tissues	Hep-2	–	–	miR-138, EZH2	PI3K/AKT	miR-138 via inhibiting the expression of EZH2 and PI3K/AKT pathway had a suppressive role in LSCC proliferation	[32]
40 pairs of LSCC and adjacent normal tissues	SNU899, SNU46	–	–	miR-375, miR-205, PTEN, E-cadherin, Vimentin, Snail2	AKT	miR-375/205 via AKT-mediated EMT could be involved in the invasion and migration of LSCC	[33]
10 pairs of LSCC and adjacent normal tissues	Hep-2, AMC-HN-8, HaCaT	–	–	miR-132, FOXO1, p21, p27, Cyclin-D1	PI3K/AKT	miR-132 by up-regulating FOXO1 and activating the PI3K/AKT pathway could act as an oncogene in LSCC cell proliferation and growth	[34]
–	D-Hep2, T-Hep2	–	–	AURKA, FAK, P130, E2F4	PI3K/AKT	AURKA via the FAK/PI3K/AKT axis could promote invasion and migration of LSCC tumor cells	[35]
–	SNU-46	–	–	DJ-1, PTEN	PI3K/AKT, mTOR	Overexpression of DJ-1 via activating the PI3K/AKT/mTOR pathway could accelerate proliferation rate, migration, and invasion of LSCC cell	[36]
85 pairs of LSCC and adjacent normal tissues	Hep-2	–	–	TSLC1, Bcl-2, p21, Caspase-3, Bax, MMP-2/9	AKT	Overexpression of TSLC1 via AKT signaling could reduce and suppress proliferation and invasiveness and induce apoptosis of LSSC cells	[37]

### Esophageal squamous cell carcinoma (ESCC)

Expression analyses esophageal cancer tissues have shown up-regulation of miR-21, PI3K, and AKT, while down-regulation of PTEN in these tissues compared with adjacent non-cancerous tissues. Notably, samples obtained from patients with lymph node metastases and poor differentiation levels had lower expression of PTEN and higher levels of PI3K and AKT proteins. Suppression of miR-21 levels in esophageal cancer cells has led to up-regulation of PTEN, down-regulation of PI3K and AKT and reduction of proliferation rate, migration, and invasion of cells. This miRNA has been found to target PTEN. Cumulatively, miR-21 has been shown to target important molecules in PTEN/PI3K/AKT signal pathway, enhancing proliferation, migration, invasiveness, and cell cycle transition, and suppressing apoptotic pathways in esophageal SCC cells [47]. Another study in esophageal SCC patients has shown correlation between p-EGFR expression and all of the other phosphorylated biomarkers. Notably, gender, N stage, and expression levels of p-AKT1 have been independently correlated with overall survival of patients. In fact, over-expression of p-AKT1 has been found to be indicative of low survival. However, levels of EGFR and p-EGFR have not been correlated with patients’ survival [48]. Moreover, dysregulation of PAFR via PI3K/AKT pathway has been reported to contribute to the progression of esophageal SCC [49].

**Fig. 1:**
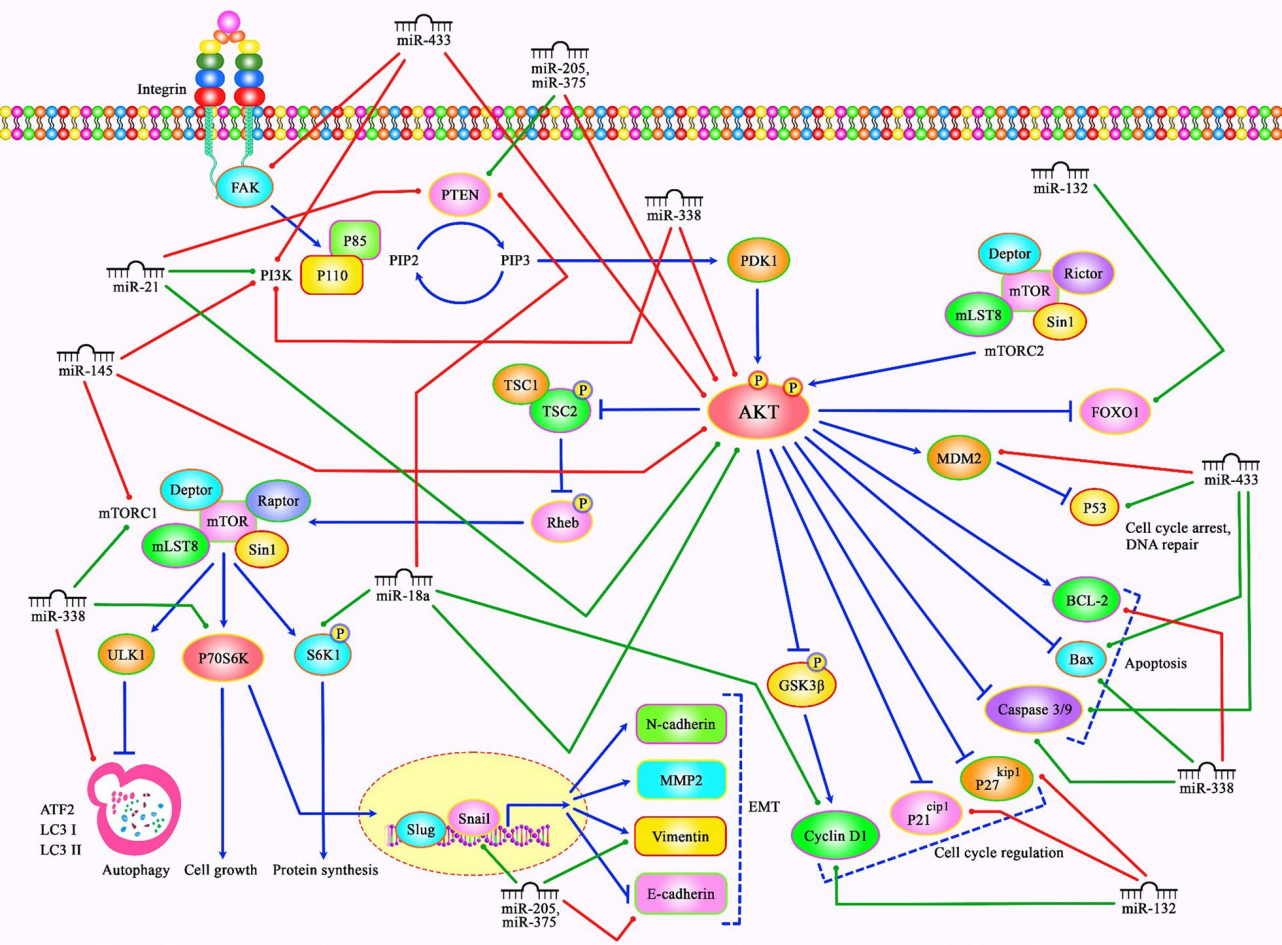
A schematic diagram of the role of several miRNAs in triggering the PI3K/AKT signaling cascade in Cervical Cancer, LSCC and ESCC. Mounting studies have revealed that dysregulation of PI3K/AKT signaling pathway can play a crucial role in the carcinogenesis especially in squamous cell carcinomas. A recent study has detected that overexpression of miR-433 could suppress the growth and metastasis of cervical cancer cells via down-regulating the FAK/PI3K/AKT signaling cascade, and could enhance the apoptosis and caspase-3/-9 function. Moreover, miR-433 could promote the expression levels of p53 and Bax, and inhibit that of MDM2 in cervical cancer [18]. Further experiment has validated that miR-132 plays an oncogenic role in laryngeal squamous cell carcinoma by suppressing the expression of FOXO1, p27, and p21. Overexpression of this miRNA could promote cell proliferation and tumor growth via up-regulating cyclin D1 as well as activating the PI3K/AKT pathway in LSCC cells [34]. Another research has pointed out that miR-21 could have a significant role in enhancing cell proliferation, migration, invasion, and cell cycle, and suppressing apoptosis of human esophageal cancer cells via down-regulating the expression of PTEN and activating PI3K/AKT signaling pathway [47]. Green lines indicate the positive regulatory effect among miRNAs and their targets, and red lines depict negative ones among them. All information regarding the role of these miRNAs involved in the modulation of PI3K/AKT signaling cascade in various types of squamous cell carcinomas can be seen in Tables 1–4

On the other hand, vitamin E succinate could induce apoptosis of esophageal SCC cells through modulation of PI3K/AKT signaling this agent has decreased growth of EC109 cells by approximately 45 and 81% in concentrations of 10 and 100 µM, respectively [50]. Moreover, Dasatinib via suppressing the PI3K/AKT and STST3 pathways could improve sensitivity to cisplatin in esophageal SCC cells [51]. Table 3 shows the role of PI3K/AKT pathway in esophageal SCC. Figure 1 illustrates the aberrant expression of various miRNAs, which contribute to adversely modulating the PI3K/AKT signaling pathway involved in triggering several kinds of squamous cell carcinomas.

**Table 3:** Role of PI3K/AKT pathway in esophageal squamous cell carcinoma

Samples	Cell lines	Drug/phytotherapy	Dose range	Target	Pathway	Function	Refs.
ESCC (n = 89), NCLM (n = 58)	TE11	–	–	miR-21, PTEN	PI3K/AKT	miR-21 through modulation of PTEN/PI3K/AKT pathway promotes invasion/migration, proliferation, cell cycle progression, and resistance to apoptosis of ESCC cells	[47]
ESCC (n = 275)	–	–	–	EGFR, ERK1/2, STAT3	AKT1	Phosphorylated AKT1 could be involved in poor prognosis in ESCC	[48]
ESCC (n = 295)	KYSE180, KYSE140, KYSE150, KYSE30, KYSE410, KYSE450, KYSE510	–	–	PAFR, c-myc, survivin, MMP2/9, VEGF		Dysregulation of PAFR via PI3K/AKT pathway could contribute to the progression of ESCC	[49]
–	EC109	Vitamin E succinate (VES)	0–100 µM	Bad, Bcl-2, Caspase-9, p70S6K, 4E-BP1,	PI3K/AKT, mTOR	VES via PI3K/AKT signaling pathway can activate apoptosis in ESCC	[50]
–	KYSE140, KYSE150, KYSE30, KYSE410, KYSE450, KYSE510	Dasatinib, cisplatin	10–500 nM, 0–15 µM	Src, c-myc, MMP-9, VEGF	PI3K/AKT, STAT3	Dasatinib via suppressing the PI3K/AKT and STST3 pathways could improve sensitivity to cisplatin in ESCC cells	[51]
-	TE13, Eca109	–	–	miR-18a, Cyclin-D1, PTEN, S6K1, pRb-S780	PI3K/AKT, mTOR	miR-18a by increasing cyclin-D1 via regulating the PTEN/PI3K/AKT/mTOR axis could promote cell proliferation of ESCC cells	[52]
nude mice, 26 pairs of ESCC and nearby non-cancerous tissues	EC109, KYSE30	–	–	Urokinase plasminogen activator (uPA), GSK-3β	PI3K/AKT, ERK	uPA realized from cancer-associated fibroblasts (CAFs) via the PI3K/AKT and ERK pathways can promote migration, invasion, and proliferation of ESCC cells	[53]
nude mice, 20 pairs of ESCC and nearby non-cancerous tissues	Eca109, TE-1, EC109, HET-1A	–	–	RUNX2, PARP, Caspase-3, GSK-3β	PI3K/AKT, ERK	Expression of RUNX2 by activating the PI3K/AKT and ERK pathways could play an oncogenic role in ESCC cells	[54]
–	KYSE-30	Aprepitant	0–100 µM	NF-kB	PI3K/AKT	SP/NK1R system via the PI3K/Akt/NF-kB pathway could be involved in the pathogenesis of ESCC	[55]
–	EC109, KYSE510, EC9706, NE2, COLO680N, SHEE, EC171, EC18, EC8712	–	–	miR-200b, E-cadherin, Vimentin, ZEB1/2	Kindlin-2/integrin β1/AKT	miR-200b via inhibiting the Kindlin-2-integrin β1/AKT pathway could decrease invasion of ESCC cells	[56]
145 pairs of ESCC and adjacent normal tissues	–	–	–	PTEN, P70S6K1, 4E-BP1	PI3K/AKT, mTOR	PTEN low expression and induction of PI3K/AKT/mTOR signaling can increase ESCC progression	[57]
ESCC (n = 68),	TE-8, TE-9, TE-15, Het-1A	–	–	CCL3,CCR5/1, MMP2, MMP9, VEGFA	PI3K/AKT, MEK/ERK	CCL3–CCR5 axis via the MEK/ERK and PI3K/AKT pathways could promote invasion, migration, and angiogenesis of ESCC cells	[58]
BALB/c nude mice	Eca109, TE-1	–	–	HPV16 E6-E7, p75NTR	PI3K/AKT	HPV16 E6-E7 via up-regulating the p75NTR and activating the PI3K/AKT pathway could act as a cancer stem-like cells (CSCs) phenotypes promoter in ESCC cells	[59]

**Table 4:** Role of PI3K/AKT pathway in pharyngeal squamous cell carcinoma

Type of diseases	Samples	Cell lines	Drug/phytotherapy	Dose range	Target	Pathway	Function	Refs.
Hypopharyngeal Squamous Cell Carcinoma (HPSCC)	HPSCC (n = 55)	FaDu	GDC-0980, Refametinib	0–5 µM, 0-20 µM	cyclin D1, p27, pRb, p-PKCζ, p-Integrin β1	PI3K/AKT, MAPK/ERK	GDC-0980 and refametinib via inhibiting the PI3K/AKT and MAPK/ERK pathways can suppress HPSCC cell proliferation, migration, and arrest cell cycle	[60]
HPSCC	16 pairs of HPSCC and nearby non-cancerous tissues	FaDu	–	–	calcium-binding protein A11, S100A11, EGFR, CD44, MMP2/9, Bcl-2	PI3K/AKT, mTOR	S100A11 via the PI3K/AKT pathway participates in the migration, carcinogenesis and protection of HPSCC from cell death induced by 5-Fu	[63]
HPSCC	12 pairs of HPSCC and adjacent normal tissues, male BALB/cAnN.Cg nude mice	FaDu	NVP-BEZ235, Cisplatin	50 nM, 2000 nM	4E-PB1, Caspase 3, PARP	PI3K/AKT, mTOR	NVP-BEZ235 when combined with cisplatin could synergistically inhibit HPSCC cell proliferation and arrest cell cycle at G2/M phase via the PI3K/AKT/mTOR pathway	[61]
HPSCC	–	FaDu	–	–	JARID1B, K10, Flag, H3K4me3, β-catenin	SHIP1/AKT	JARID1B via the SHIP1/AKT pathway could improve HPSCC cell differentiation and suppress proliferation	[62]
HPSCC	56 pairs of HPSCC and adjacent normal tissues, male nude mice	FaDu	–	–	Argonaute 2 (AGO2), p53, Caspase-3, FAK	PI3K/AKT	AGO2 via the FAK/PI3K/AKT pathway could increase tumor growth, proliferation, migration, and invasion of HPSCC cell	[64]
HPSCC	–	FaDu, 293 T	EGFRmAb–AuNPs	20 mM	Bcl-2, Bax, Caspase-3/9	PI3K/AKT, mTOR	Photothermal treatment with EGFRmAb–AuNPs via the PI3K/AKT/mTOR pathway and DNA destruction enhances apoptosis in HPSCC cells	[65]
Oropharyngeal Squamous Cell Carcinoma (OPSCC)	OPSCC (n = 116)	–	–	–	PTEN	AKT	HPV could activate the PI3K/AKT pathway and increase levels of pAKT (Ser473) and PTEN in OPSCC	[66]
OPSCC	OPSCC (n = 121)	–	–	–	EGFR, PTEN	AKT	Because of HPV, level of PTEN, EGFR and pAKT, could be different between oropharyngeal and oral cavity squamous cell carcinoma	[67]
Pharyngeal Squamous Cell Carcinoma (PSCC)	–	NHOK, FaDu	Adenosine	0–3 mM	Bax, Bcl-2, caspase-3/9	PI3K/AKT, mTOR	Adenosine via the PI3K/AKT/mTOR pathway and activating caspase-3/9 could induce mitochondrial intrinsic apoptosis in PSCC cells	[68]

### Pharyngeal squamous cell carcinoma (PSCC)

In patients with hypopharyngeal SCC, expression of p-Akt and p-Erk has been shown to be remarkably elevated parallel with progression of clinical stage, indicating the possible roils of PI3K/Akt and MAPK/ERK pathways in evolution and progression of this type of cancer. Notably, GDC-0980 and Refametinib have exerted cytotoxic effect on hypopharyngeal SCC cells. These agents could block cell cycle progression in G1 phase, reduce cyclin D1 and p-Rb levels and increase p27 levels. GDC-0980 could also inhibit migratory potential of these cells and reduce levels of p-PKCζ, p-Integrin β1 and uPA metastasis-related proteins. Taken together, dual suppression of PI3K/Akt and MAPK/ERK pathways by mentioned agents can be regarded as a possible strategy for treatment of hypopharyngeal SCC [60]. NVP-BEZ235 when combined with cisplatin could inhibit proliferation of hypopharyngeal SCC cells and arrest cell cycle at G2/M phase via modulation of the PI3K/AKT/mTOR pathway [61].

JARID1B, as a tumor suppressor, via the SHIP1/AKT pathway could improve differentiation of hypopharyngeal SCC cells and suppress their proliferation [62]. On the other hand, S100A11 could play an important role in the migration, carcinogenesis and protection of HPSCC from cell death induced by 5-Fu via the PI3K/AKT pathway [63]. Table 4 shows the role of PI3K/AKT pathway in pharyngeal squamous cell carcinoma.

### Oral squamous cell carcinoma (OSCC)/tongue squamous cell carcinoma (TSCC)

Lycopene has been revealed to inhibit proliferation, migration and invasiveness of oral SCC cells as well as in vivo growth of tumors. Moreover, this substance could suppress epithelial–mesenchymal transition and activate apoptotic pathways through decreasing activity of PI3K/AKT/mTOR signaling. These effects are exerted through enhancing expressions of E-cadherin and Bax and decreasing levels of N-cadherin, p-PI3K, p-AKT, p-m-TOR, and bcl-2 [69]. Thymoquinone has also been shown to suppress invasion, proliferation and migration of oral SCC cells and induce their apoptosis via inhibiting the PI3K/AKT pathway [70]. Moreover, Licochalcone A could suppress migration, invasion, and proliferation of oral SCC cells via modulation of the PI3K/AKT pathway [71].

A number of non-coding RNAs have been reported to exert their effects in the pathogenesis of oral SCC through modulation of this pathway. This speculation has been verified by knock-down experiments. For example, suppression of lncRNA MALAT1 could inhibit invasion, migration, and proliferation of TSCC cells via suppressing the PI3K/AKT pathway and down-regulating MMP-9 [72]. Moreover, circCDR1 has been shown to improve the viability of oral SCC cells by promoting autophagy via the AKT/ERK/mTOR pathway [73]. Table 5 shows the role of PI3K/AKT pathway in oral SCC.

**Table 5:** Role of PI3K/AKT pathway in oral squamous cell carcinoma

Samples	Cell Lines	Drug/ phytotherapy	Dose range	Target	Pathway	Function	Refs.
Male Balb/c nude mice	CAL-27, SCC-9	Lycopene	0–2 µM	Bax, Bcl-2, E-cadherin, N-cadherin	PI3K/AKT, mTOR, EMT	Lycopene by suppressing the EMT pathway and activating the PI3K/AKT/mTOR pathway could induce apoptosis and inhibit invasion, cell proliferation, and migration of OSCC cells	[69]
–	KB, K562, MCF-7	Thymoquinone (TQ)	0–2 µM	–	PI3K/AKT	TQ through suppression of the PI3K/AKT pathway could suppress invasion, proliferation, migration and induce apoptosis in OSCC cells	[70]
TSCC (n = 72 patients)	SCC4, SCC15, SCC25, Hs 680	–	–	LncRNA MALAT1, MMP-9	PI3K/AKT	Suppression of lncRNA MALAT1 could inhibit invasion, migration, and proliferation of TSCC cells via suppressing the PI3K/AKT pathway and down-regulating MMP-9	[72]
52 OSCC tissues with the corresponding non-tumor tissues	CGHNK2, SCC25, HSC3	–	–	FBXW7, miR-27a, Vimentin, N-cadherin, E-cadherin	PI3K/AKT	Up-regulation of FBXW7 and downregulation of miR-27a via the PI3K/AKT pathway can suppress the proliferation and cell growth of OSCC	[74]
OSCC (n = 80), adjacent non-tumor tissues (n = 7)	HUVEC, CAL27	–	–	miR-210-3p, EFNA3	PI3K/AKT	miR-210-3p by increasing the phosphorylation rate of AKT could promote OSCC cells angiogenesis, migration, and proliferation	[75]
Datasets	HO1-N-1, SCC-9, HNOEC	–	–	ITGA5, ERK	PI3K/AKT	ITGA5 via the PI3K/AKT pathway could play an oncogenic role and promote invasion, proliferation, and migration of OSCC cells	[76]
57 pairs of OSCC and adjacent non-tumor tissues, female BALB/c nude mice	Tca-8113, SCC-15, HOK			CircCDR1, HIF-1α, p62, LC3I/II, ATG5, Bax, Bcl-2, Caspase-3	AKT, ERK1/2, mTOR	CircCDR1 via the AKT/ERK/mTOR pathway could improve the viability of OSCC cells by promoting autophagy	[73]
BALB/c nude mice	SCC4, CAL-27	Licochalcone A	0–100 μM	PCNA, MMP-2/9	PI3K/AKT	Licochalcone A could suppress OSCC cells migration, invasion, and proliferation via modulation of the PI3K/AKT pathway	[71]
98 paraffin embedded OSCC samples	HSC3, OSCC3, SCC4, SCC7, Cal27, HaCaT	–	–	SPARC, PDGFB, PDGFRβ	PI3K/AKT	SPARC via the PI3K/AKT/PDGFB/PDGFRβ axis could promote metastasis and proliferation of OSCC cells	[77]
male Syrian hamsters	SCC131, SCC4	Astaxanthin (AXT), wortmannin, Bay-11, S31-201	0–1200 μM, 0–200 nM, 0–10 μM, 0–120 μM	NF-kΒ, Bcl-2, Bax, Cyclin-D1, p21, MMP-2/9, Caspase-3/9, HIF-1α, VEGF, VEGFR2	PI3K, STAT3	AXT in combination with wortmannin, Bay-11 or S3I-201 via the PI3K/NF-kΒ/STAT3 axis could suppress apoptosis evasion, invasion, proliferation, and angiogenesis of OSCC cells	[78]
124 pairs of paraffin-embedded OSCC and adjacent tissues, female BALB/c mice	SCC15, SCC25	–	–	TGF-β, SOX2, BMI1, ERK1/2, ABCG2, CD44, IVL	AKT/FOXO3a	TGF-β via AKT/FOXO3a axis could induce stemness in OSCC	[76]
62 pairs of OSCC and adjacent non-tumor tissues, female BALB/c nude mice	SCC25, Cal27	–	–	B7-H3, PFKFB3, Glut1	PI3K/AKT, mTOR	Protein B7-H3 via the PI3K/AKT/mTOR pathway could increase tumor glucose uptake, aerobic glycolysis and metastasis in OSCC	[73]
20 pairs of OSCC and adjacent non-tumor tissues, athymic nude mice	SCC9 SCC15, SCC25, CAL27, hTERT-OME	Tanshinone IIA	0–5 μM	HK2/1, VDAC1, Bax, GSK-3β, Caspase-3, PARP	AKT/c-Myc	Tanshinone IIA via the AKT/c-Myc pathway could inhibit OSCC by reducing of glycolysis	[74]
TSCC (n = 40)	Cal 27, SCC9	–	–	miR-21-5p, Bax, Bcl-2, PDCD4, FOXO1	PI3K/AKT	Downregulation of miR-21-5p by targeting PDCD4 that knockdown the PI3K/AKT/FOXO1 pathway could inhibit the invasion and proliferation of TSCC	[79]
BALB/c nu/nu nude mice	HOMEC, TSCCA, SCC15, CAL27	–	–	Per2, LC3B, p62, Beclin-1	PI3K/AKT, mTOR	Per2 via the PI3K/AKT/mTOR pathway can inhibit OSCC progression by activating autophagy	[80]
50 sample of OSCC and 10 of adjacent non-tumor tissues, female athymic nude mice	CAL27, HSC4, SCC15, 293 T	–	–	USP13, GLUT1, HK2	PTEN/AKT	USP13 via regulating PTEN/AKT pathway act as a tumor suppressor	[81]
Male Balb/c‐nude mice	TSCCa, Tca‐8113			CCN5, Bax, Cyclin-D1/E, CDK2, Bcl-2, Procaspase-3/9	PI3K/AKT	CCN5 via the PI3K/AKT pathway can suppress proliferation and promote apoptosis of OSCC	[82]
116 pairs of OSCC and adjacent non-tumor tissues	SCC-25, HOK	–	–	PAR-2	PI3K/AKT, mTOR	PAR-2 via the PI3K/AKT pathway could enhance invasion, migration, and proliferation of OSCC cells	[83]
BALB/c nude mice	SCC 4, HSC3, CAL27, HN6, HOK	–	–	TROP2, PTEN, PDK1	PI3K/AKT	TROP2 via PI3K/AKT could promote cell growth, migration, proliferation, and invasion in OSCC cells	[84]
–	OSC‑4	–	–	GSK-3β Rab-5B, Calnexin, Cytochrome-c	AKT	Macrophage-derived exosomes by activating the AKT/GSK-3β pathway could reduce sensitivity to chemotherapeutic agents in OSCC cells	[85]
OSCC (n = 155)	OC3, OECM1, SCC4, SCC25, SAS, CGNHC9	–	–	Activin A, EGFR, SP1, Smad-2/3/4	PI3K/SP1	Activin A via activating the PI3K/SP1 pathway could regulate EGFR was necessary for the carcinogenesis of OSCC	[86]
–	SCC131	Syringic acid (SRA)	0–45 μm/mL	TNF-α, COX-2, iNOS, IL-6, VEGF, NF-κB	PI3K/AKT	SRA via suppression of the PI3K/AKT/NF**-**κB axis could induce disruption of MMP, mitochondrial apoptosis, and inhibit cell proliferation and migration	[87]
–	SCC-25	–	–	Alpha-hederin (α-HN), Bax. Bcl-2	PI3K/AKT, mTOR	α-HN via the PI3K/AKT/mTOR pathway can inhibit cell proliferation, adhesion, invasion and induce apoptosis of OSCC cells	[88]
OSCC (n = 53), BALB/C nude mice	Cal-27, SCC-25, HIOEC	–	–	miR-210-3p, EFNA3, N-cadherin, E-cadherin	PI3K/AKT	Up-regulation of miR-210-3p via the Ephrin-A3/PI3K/AKT pathway could inhibit OSCC cells development and metastasis	[89]
162 OSCC samples with oral submucous fibrosis (OSF), 38 normal buccal mucosa (NBM)	CAL27, HN6, UM1, SCC9, HOK, 293 T	–	–	circEPSTI1, miR-942-5p, LTBP2, Vimentin, N-cadherin, E-cadherin	EMT, PI3K/AKT, mTOR	The circEPSTI1/miR-942-5p/LTBP2 axis via the EMT and PI3K/AKT/mTOR pathways could promote invasion, migration, and proliferation of OSCC cells	[90]
Male BALB/c nude m	NHOK, SCC-25, SCC-9	Genipin	0–400 μM	Survivin, PARP, Caspase-3, p62, Beclin-1, LC3II/LC3I	PI3K/AKT, mTOR	Genipin via the PI3K/AKT/mTOR pathway could induce autophagy and apoptosis and suppress the proliferation of OSCC cells	[91]
–	CAL-27, SCC-25	Pristimerin, 5-fluorouracil, cisplatin	0–1 μM, 0–150 μM, 0–20 μM	p21, p27, p53 PARP, Caspase-3,	AKT, MAPK/ERK	Pristimerin via the MAPK/ERK1/2 and AKT pathways could induce apoptosis and suppress proliferation of OSCC cells more than cisplatin and 5-fluorouracil	[92]
–	HOEC, HN4, HN30, HN6	–	–	PLAC8, PCNA, c-Myc, GSK-3β, Cyclin-D1, E-cadherin, Vimentin	Wnt/β-catenin, PI3K/AKT	PLAC8 via the PI3K/AKT/GSK-3β and Wnt/β-catenin pathways could inhibit carcinogenesis and EMT of OSCC cells	[93]
Cohort, BALB/c nu/nu female nude mice	SCC15, CAL27, HOMEC, TSCCA	–	–	lncRNA CASC9, p62, Bcl-2, Bax, LC3II/LC3I	AKT/mTOR	LncRNA CASC9 via the AKT/mTOR pathway can promote tumoral cell proliferation and progression by suppressing autophagy in OSCC	[94]
86 sample of OSCC and 32 sample of adjacent non-tumor tissues, SPF-grade BALB/c nu/nu female nude mice	HOMEC, TSCCA, SCC15, CAL27	–	–	Per1, LC3BII/I, Beclin-1, P62, Bax	AKT/mTOR	Clock gene Per1 via the AKT/mTOR pathway could suppress autophagy and improve cell proliferation in OSCC	[95]
OSCC (n = 92)	SCC4, Cal27, HSC3, HaCaT	–	–	LGALS3BP	PI3K/AKT	LGALS3BP via the PI3K/AKT pathway could promote migration and proliferation of OSCC cells	[96]
–	CAL27, SCC9	–	–	Notch1, N‐cadherin, Vimentin, E‐cadherin, β‐catenin, P21, Cyclin-D3	EGFR, PI3K/AKT	Membrane-tethered Notch1 via activating the EGFR/PI3K/AKT axis could exhibit oncogenic property in OSCC	[97]
BALB/cnu/nu nude mice	Cal27	–	–	miR-134, LAMC2, GSK-3β, Caspase-9, Bcl-2, Bax	PI3K/AKT	miR-134 by down-regulating LAMC2 via the PI3K/AKT pathway can suppress cell migration, invasion, and metastases of OSCC cells	[98]
OSCC (n = 134)	SNU1041, SCC25, SCC4, SCC9, hNOK	–	–	lncRNA FTH1P3, GSK-3β	PI3K/AKT, Wnt/β-catenin	LncRNA FTH1P3 via the PI3K/AKT/GSK-3β/ Wnt/β-catenin axis could promote migration and invasion in OSCC cells	[99]
8 pairs of OSCC and adjacent normal tissue	SCC-9, TEC, SCC-25, TSCCa, Tca-8113	–	–	miR-194, FoxO3a, Cyclin-D1, p21, AGK	PI3K/AKT	miR-194 by reducing the PI3K/AKT/FoxO3a axis could inhibit cell proliferation of OSCC	[100]
OSCC (n = 125)	–	–	–	PTEN	PI3K/AKT, mTOR	Activity of PI3K/AKT pathway is enhanced in the gingival, hard palate, and alveolar ridge SCC. The expression of p-mTOR could be considered as a biomarker of survival in OSCC	[101]
8 pairs of OSCC and normal oral mucosal tissue	–	–	–	c-Met	PI3K/AKT	Carcinoma associated fibroblasts (CAF) via the c-Met/PI3K/AKT pathway could induce lymphangiogenesis in OSCC	[102]
OSCC (n = 56), BALB/c-nude mice	CAL27, SCC9, HCT 116, 293 T	Cisplatin	0–35 µg/mL	miR-22, KAT6B, Caspase-3, PARP, p53, Bcl-2, NF-kB	PI3K/AKT	Overexpression of miR-22 and suppression of KAT6B via the PI3K/AKT/NF-kB pathway can increase the OSCC cells apoptosis by enhancement of the sensitivity of these cells to cisplatin	[103]
–	KB	Sanguinarine	0–2 µM	Caspase-3/8/9, Fas/FasL, Bid, Bax, Bcl-2, TRAIL	PI3K/AKT	Sanguinarine via suppressing the PI3K/AKT pathway could induce apoptosis of OSCC cells	[104]
Paraffin-embedded OSCC (n = 90)	–	–	–	GSK-3β	AKT, mTOR	AKT and mTOR proteins could be involved in OSCC progression and modulate the biology of OSCC. In addition, GSK-3β could regulate the mechanism of OSCC dissemination to the cervical lymph node	[105]
–	SCC25, 1483, FeOSCC, K9OSCC	doxorubicin, AD198	0–1 µM, 0–1 µM	cPARP, ERK1/2, p38, GSK-3β, Caspase-3/7	PI3K/AKT	Dox or AD198 as an anthracycline therapy via inhibiting the PI3K/AKT can inhibit cell proliferation in OSCC cells	[106]
OSCC (n = 12), male nude BALB/c mice	SCC4, SCC25, OML1, OML1-R	–	–	Bax, Caspase-3, Cyclin-D1, CDK4	PI3K/AKT, mTOR	The PI3K/mTOR pathway is invovled in sensitizing OSCC cells to radiotherapy	[107]
OSCC (n = 25), adjacent non-tumor tissues (n = 5), nude mice	Tca-8113, KB	–	–	Zinc Finger Protein 703, c-Myc, GSK-3β, Vimentin, Snail, N-cadherin, E-cadherin	PI3K/AKT	Zinc Finger Protein 703 via PI3K/AKT/GSK-3β pathway could promote metastasis and cell proliferation of OSCC	[108]
60 pairs of OSCC and adjacent normal tissue	SCC-25, HSC3	–	–	Cyclin-D1, T-cadherin	PI3K/AKT, mTOR	T-cadherin via inhibiting the PI3K/AKT/mTOR pathway could suppress the proliferation of OSCC	[109]
Male Syrian hamsters	Cal27, LN4, Leuk1	Salvanic acid B	0–600 µM	GLUT1, HIF-1α	PI3K/AKT	Salvanic acid B via the PI3K/AKT/HIF-1α axis could suppress OSCC malignant transformation by inhibiting aberrant glucose metabolism	[110]
58 pairs of TSCC and adjacent normal tissue	SCC9, SCC25	–	–	FoxM1, E-cadherin, Vimentin	c-Met/AKT	FoxM1 via the c-Met/AKT-dependent positive feedback loop pathway could promote EMT, migration, and invasion of TSCC	[111]
female BALB/c nude mice	SCC-25, UM1, UM2, HSC-3, Cal 27	Oridonin	0–10 mg/kg	Bcl-2, Bax, Caspase-3/9, Cyclin-D1/D3, p21	PI3K/AKT	Oridonin through suppression the PI3K/AKT pathway could suppress proliferation and induce apoptosis and G2/M-phase arrest in OSCC cells	[112]
–	SCC25	Plumbagin (PLB)	0–5 µM	Bax, Bcl-2, Caspase-3/9, GSK-3β, Beclin-1, LC3-I/II	p38 MAPK, PI3K/AKT, mTOR	Plumbagin via MAPK and PI3K/AKT/mTOR-mediated pathways could promote autophagy, G2/M arrest, apoptosis, and increase intracellular levels of ROS in TSCC cells	[113]
Female BALB/c mice, 36 pairs of OSCC tissues and adjacent normal tissues	TSC-15, CAL27, TSCCa, Tca8113, SCC-4, SCC-25	–	–	PON3, AP-1	PI3K/AKT	PON3 via the PI3K/AKT pathway can promote migration, invasion, and cell proliferation in OSCC cells	[114]

### Unidentified types of head and neck squamous cell carcinoma (HNSCC)

Expression of FKBP9P1 has been shown to be increased in HNSCC samples and cells. Over-expression of this gene has been correlated with advanced T, N and clinical stages as well as poor prognosis of affected individuals. FKBP9P1 silencing has suppressed proliferation, migratory potential, and invasiveness of these cells, possibly through inhibition of PI3K/AKT signaling [38]. PFN2 is another up-regulated gene in HNSCC and cells. PFN2 silencing has suppressed proliferation, invasiveness, and migratory potential of HNSCC cells, possibly through reduction of Akt and GSK-3β phosphorylation as well as decrease in β-catenin levels. In other words, PFN2 has been shown to promote proliferation and metastatic ability of HNSCC through inducing activity of the PI3K/Akt/β-catenin pathway [39]. Similarly, DKK3 has been shown to increase the malignant properties of HNSCC via the PI3K/AKT/mTOR and MAPK pathways [40].

An in vitro study has shown that the anti-cancer agent osthole induces cell cycle arrest at G2/M phase and blocks proliferation of HNSCC cells via suppressing the PI3K/AKT pathway [41]. Finally, PI3K/AKT pathway has been shown to mediate the adaptive resistance to anti-programmed death-1 (PD1) therapy through upregulating Tim-3 [42]. Table 6 shows the role of PI3K/AKT pathway in head and neck squamous cell carcinoma.

**Table 6:** Role of PI3K/AKT pathway in head and neck squamous cell carcinoma

Samples	Cell Lines	Drug/ phytotherapy	Dose range	Target	Pathway	Function	Refs.
–	SCC-4, SCC-9, SCC-25, FaDu, UM-SCC-22A	Chloroquine (CQ)	0–30 µM	MAP1LC3B, SQSTM1	PI3K/AKT, mTOR	PI3K/AKT/mTOR autophagy pathway could be blocked by CQ that had an inhibitory effect on HNSCC proliferation	[43]
114 pairs of HNSCC and adjacent normal tissues	FaDu, Cal-27, SCC4, SCC9, HaCaT	–	–	RNA FKBP9P1	PI3K/AKT	Silencing expression of RNA FKBP9P1 via PI3K/AKT signaling pathway can constrain the progression of HNSCC	[38]
–	Fadu, SSC-9, SSC-25, OSC-19, Cal-27, HOK	–	–	Profilin 2 (PFN-2), GSK-3β, β-catenin	PI3K/AKT	PFN2 via activating the PI3K/AKT/β-catenin pathway could promote the proliferation and metastasis of HNSCC	[39]
–	FaDu, Cal27, SCC25, HN4	Osthole	0–240 µM	PTEN, Cdc2, Cyclin-B1, Bcl-2, Bax, PARP1, Survivin, Caspase3/9	PI3K/AKT	Osthole via suppressing the PI3K/AKT pathway could have an anti-tumor effect on HNSCC	[41]
Male BALB/cAJcl-nu/nu nude mice	HSC-3 shDKK3, HSC-3 shScr	–	–	DKK3, β-catenin, GSK-3β, p55, PDK1, p38, TGF-β	PI3K/AKT, mTOR, MAPK	DKK3 via the PI3K/AKT/mTOR and MAPK pathways could increase the malignant properties of HNSCC	[40]
Female BALB/C nude, HNSCC (n = 298), NCLM (n = 98)	FaDu, 293 T, AMC-HN-8, Tca-8113, Cal-27	–	–	STC2, Snail, Vimentin, E-cadherin	PI3K/AKT	STC2 via the PI3K/AKT/Snail pathway can promote HNSCC metastasis, proliferation, and tumoral cell growths	[44]
–	OSC-20, HEEC, SNU-1076, HSC-3, Ca9-22	–	–	HPIP	PI3K/AKT	Knockdown of HPIP via suppressing the PI3K/AKT pathway can inhibit invasion, proliferation, and invasion of HNSCC	[45]
HNSCC (n = 36), Female C57BL/6 mice	PBMC, TIL	–	–	Tim-3	PI3K/AKT	Adaptive resistance to anti-programmed death-1 (PD1) therapy through up-regulating Tim-3 could be mediated via the PI3K/AKT pathway	[42]
HNSCC (n = 36)	Tu686, 5-8F	–	–	Metadherin (MTDH), VEGF	PI3K/AKT	MTDH could regulate VEGF expression via the PI3K/AKT pathway, resulting in HNSCC metastasis and angiogenesis	[46]

## Cutaneous SCC

α-mangostin has been shown to suppress skin tumor formation and growth, decrease levels of pro-inflammatory molecules and increase levels of anti-inflammatory ones both in tumor and circulation. Notably, this substance could induce autophagy of skin cancer cells and regulate expression of autophagy-related proteins. Most notably, α-mangostin can inhibit activity of the PI3K/AKT/mTOR signaling, as demonstrated by down-regulation of p-PI3K, p-Akt and p-mTOR [115]. Moreover, Lapatinib could suppress epithelial-mesenchymal transition in skin SCC via modulation of WNT/ERK/PI3K/AKT axis [116]. The anti-cancer effects of Lactucopicrin in skin cancer is also mediated through modulation of PI3K/AKT/mTOR pathway [103].

**Fig. 2:**
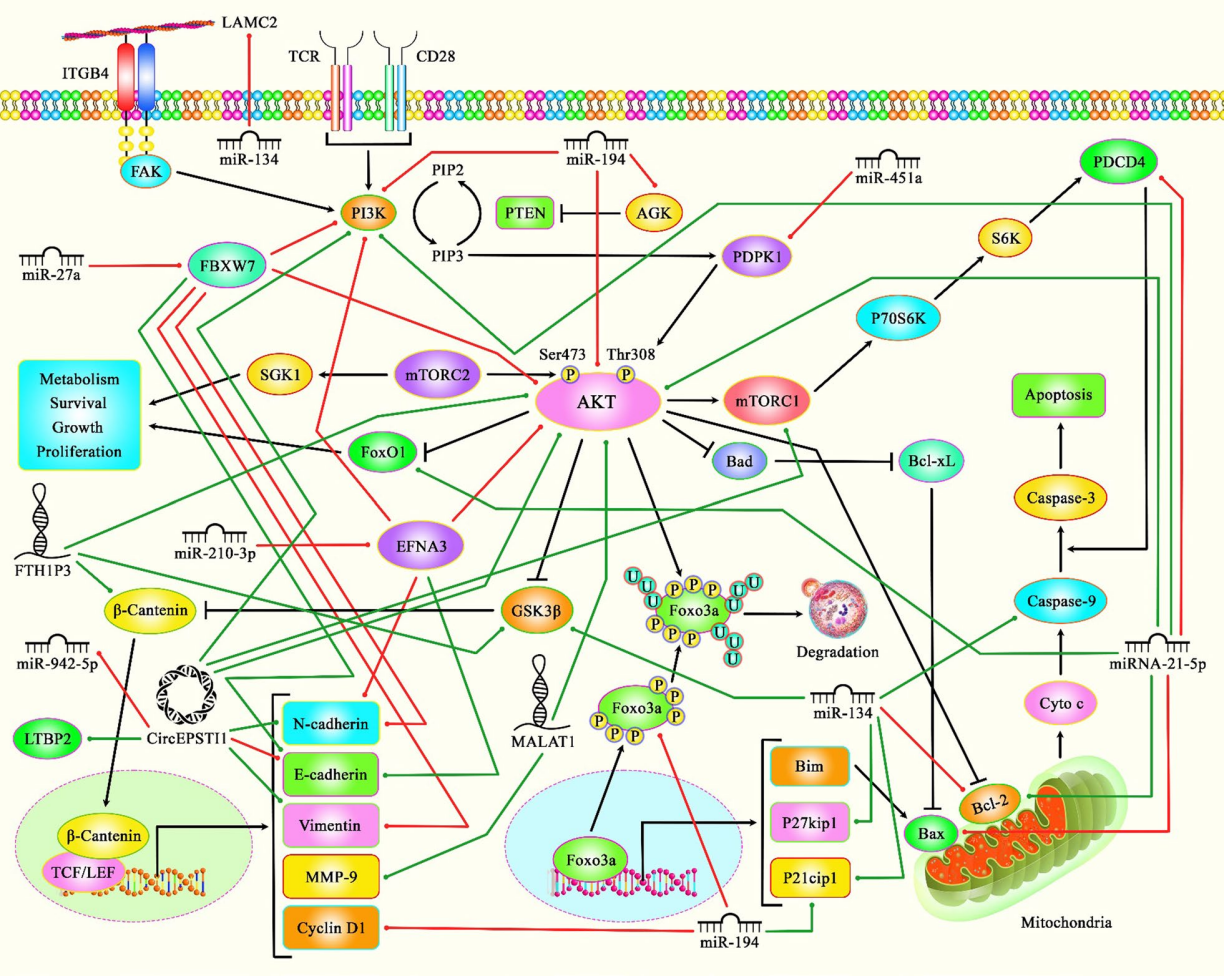
A schematic representation of the role of several ncRNAs in regulating the PI3K/AKT/mTOR signaling pathway in OSCC, TSCC and Cutaneous SCC. Accumulating evidence has revealed that various ncRNAs (lncRNAs, circRNAs, and miRNAs) could be negatively involved in triggering different kinds of SCCs via activating PI3K/AKT/mTOR signaling cascade. As an illustration, previous study has authenticated that up-regulation of lncRNA MALAT1 could promote the proliferation, migration, and invasion of tongue cancer cells via increasing the expression levels of AKT and MMP-9 [72]. Another finding confirms that overexpression of miR-21-5p could inhibit apoptosis via down-regulating the expression levels of PDCD4 as well as pro-apoptotic protein Bax and up-regulating FOXO1 and Bcl2 through directly activating the PI3K/AKT pathway in tongue squamous cell carcinoma [79]. Furthermore, mounting research has demonstrated that miRNA‑451a via directly targeting PDPK1 could suppress cutaneous squamous cell carcinoma development by modulating the PI3K/AKT signaling pathway [117]. Green lines indicate the positive regulatory effect among ncRNAs and their targets, and red lines depict negative one among them. All the information regarding the role of these ncRNAs involved in the regulation of the PI3K/AKT signaling pathway in several kinds of squamous cell carcinomas can be seen in Tables 6, 7

A number of non-coding RNAs can also modulate progression of skin SCC through influencing activity of PI3K/AKT pathway. For instance, miR-451a via PDPK1-mediated PI3K/AKT modulation could prevent progression of skin SCC [117]. Moreover, lncRNA LINC00520 via inactivating the PI3K/AKT pathway by downregulating EGFR could prevent the progression of this type of cancer [118]. Table 7 shows the role of PI3K/AKT pathway in skin SCC. Figure 2 represents the role of several ncRNAs in various types of SCCs via regulating the PI3K/AKT/mTOR signaling pathway.

**Table 7:** Role of PI3K/AKT pathway in cutaneous squamous cell carcinoma

Type of Diseases	Samples	Cell Lines	Drug/ phytotherapy	Dose range	Target	Pathway	Function	Refs.
Skin cancer	Female ICR mice	–	a-mangostin	5 and 20 mg/kg	IL-4/10/18, IL-1β, Bax, Caspase-3, Bcl-2, LC3-II/I, Beclin-1	PI3K/AKT, mTOR	a-Mangostin by regulating PI3K/AKT/mTOR pathway could inhibit DMBA/TPA-induced skin cancer	[115]
Cutaneous squamous cell carcinoma (CSCC)	–	SCC, A431	Lapatinib	0–5 μM	Caspase-8, Bcl-2, EGFR, N-cadherin, Vimentin	WNT/β-catenin, PI3K/AKT, mTOR, ERK1/2	Lapatinib via the WNT/ERK/PI3K/AKT axis could suppress EMT	[116]
Skin cancer	–	SKMEL-5	Lactucopicrin	0–30 μM	Bax, Bcl-2	PI3K/AKT, mTOR	Lactucopicrin via inhibiting the PI3K/AKT/mTOR pathway exerted anticancer effects on skin cancer cells	[103]
Skin carcinoma	–	A549, A431, PaCa-2, PC-3, MCF-7, SNU-5, HTB-39	caffeic acid n-butyl ester (CAE)	0–40 μM	Bax, Bcl-2	PI3K/AKT, mTOR	CAE via induction of apoptosis and inhibition of the PI3K/AKT/mTOR pathway could reduce proliferation of skin cancer cells	[119]
CSCC	–	HaCaT, cSCC, A431, HSC-5, SCC-12, SCL-1	–	–	miR-451a, PDPK1	PI3K/AKT	miR-451a via PDPK1-mediated PI3K/AKT modulation could prevent CSCC progression	[117]
CSCC	Female nude mice	cSCC, A431	–	–	LINC00520, EGFR, VEGF, MMP-2/9	PI3K/AKT	lncRNA LINC00520 via inactivating the PI3K/AKT pathway by decreasing EGFR could prevent the progression of CSCC	[118]
CSCC	CSCC tissues (n = 11), normal skin tissues (n = 4)	cSCC, NHEK HaCaT, A431, SCL-1	–	–	Kynureninase (KYNU)	PI3K/AKT	Downregulation of KYNU could restrain CSCC proliferation and repress the PI3K/AKT pathway	[120]
CSCC	–	SCC13, A431	High mobility group box 1 (HMGB1)	0–100 ng/mL	p42/44, p38	PI3K/AKT, MAPK	HMGB1 via the PI3K/AKT and MAPK pathways can influence tumor metastasis	[121]

## Discussion

PI3K/AKT has essential roles in the development of different types of SCC. Over-expression of PI3K, AKT, and p-mTOR has been reported in SCC tumors in association with down-regulation or absence of PTEN [122]. Gain of function mutations in constituents of this pathway, amplification of PIK3CA and AKT, overexpression of AKT and inactivating mutations or loss of PTEN are involved in the aberrant activity of this signaling pathway and subsequent progression of cancer {Simpson, 2015 #155}. Thus, identification of the underlying mechanism of over-activation of PI3K/AKT pathway in SCC has practical significance in design of novel therapeutic options.

Moreover, a number of anti-cancer drugs such as cisplatin, LY294002, Licochalcone A, Mukonal, Dehydrocostus Lactone, Curcumin, Chloroquine, Osthole, Vitamin E succinate, Dasatinib, Tanshinone IIA, Genipin, Pristimerin, 5 fluorouracil, Sanguinarine, doxorubicin, AD198, Salvanic acid B, Oridonin, Plumbagin, a-mangostin, Lapatinib, Lactucopicrin and caffeic acid n-butyl ester have been found to exert their therapeutic effects in SCC via modulation of this pathway. It is worth mentioning that drug-loaded nanospheres and microspheres as a novel strategy for drug delivery can be used to form a material, mechanism, and cell combination that can not only treat the disease, but also verify the pathway. The possibility of using these systems for delivery of afore-mentioned drugs should be studies in future studies.

In brief, the bulk of evidence shows the impact of dysregulation of PI3K/AKT pathway in the pathogenesis of SCC and determination of survival of patients with this type of cancer. Moreover, targeted therapies against this pathway have been found to be effective in reduction of tumor burden both in animal models and clinical settings. Since this pathway has an established role in the induction of epithelial-mesenchymal transition, these therapies are expected to affect tumor metastasis as well. Besides, therapeutic modalities against PI3K/AKT might act in a synergic manner with other anti-cancer modalities, enhancing the survival of affected individuals. PI3K/AKT pathway can also act as a mediator of HPV-induced cancer stem-like cells features in SCC [59], further highlighting the importance of this pathway in malignant features of SCC.

Finally, a number of molecules that regulate PI3K/AKT pathway can be used as diagnostic markers for different types of SCCs.

Recent studies have also indicated the impact on non-coding RNAs in the regulation of PI3K/AKT pathway in different cancers, including SCC [123]. Thus, when designing novel therapeutic options against this pathway, it is necessary to consider the regulatory roles of these transcripts and their expression levels in these patients. Such approach may lead to establishment of a more effective personalized therapeutic strategy.

## Data Availability

The analyzed data sets generated during the study are available from the corresponding author on reasonable request.
